# Guidelines for clinical evaluation of chronic kidney disease in early stages: AMED research on regulatory science of pharmaceuticals and medical devices

**DOI:** 10.1007/s10157-024-02514-6

**Published:** 2024-07-06

**Authors:** Yuka Sugawara, Eiichiro Kanda, Takayuki Hamano, Seiji Itano, Hirokazu Okada, Koji Tomori, Yusuke Watanabe, Wataru Asakura, Yoshitaka Isaka, Kunitoshi Iseki, Tomoko Usui, Yusuke Suzuki, Mototsugu Tanaka, Rimei Nishimura, Kei Fukami, Kunihiro Matsushita, Jun Wada, Hirotaka Watada, Kohjiro Ueki, Naoki Kashihara, Masaomi Nangaku, Research Working Group for Establishing Guidelines for Clinical Evaluation of Chronic Kidney Disease

**Affiliations:** 1 Division of Nephrology and Endocrinology, The University of Tokyo, 7-3-1 Hongo, Bunkyo-ku, Tokyo, 113-8655 Japan; 2 Medical Science, Kawasaki Medical School, Okayama, Japan; 3 Department of Nephrology, Nagoya City University Graduate School of Medical Sciences, Aichi, Japan; 4 Department of Nephrology and Hypertension, Kawasaki Medical School, Okayama, Japan; 5 Department of Nephrology, Saitama Medical University, Saitama, Japan; 6 Office of New Drug I, Pharmaceuticals and Medical Devices Agency (PMDA), Tokyo, Japan; 7 Department of Nephrology, Osaka University Graduate School of Medicine, Osaka, Japan; 8 Clinical Research Support Center, Nakamura Clinic, Okinawa, Japan; 9 Department of Nephrology, Faculty of Medicine, Juntendo University, Tokyo, Japan; 10 Clinical and Translational Research Center, Niigata University Medical and Dental Hospital, Niigata, Japan; 11 The Jikei University School of Medicine, Jikei University, Tokyo, Japan; 12 Division of Nephrology, Department of Medicine, Kurume University School of Medicine, Fukuoka, Japan; 13 Department of Epidemiology, Johns Hopkins Bloomberg School of Public Health, Maryland, USA; 14 Department of Nephrology, Rheumatology, Endocrinology and Metabolism, Okayama University Graduate School of Medicine, Dentistry and Pharmaceutical Sciences, Okayama, Japan; 15 Department of Metabolism and Endocrinology, Juntendo University Graduate School of Medicine, Tokyo, Japan; 16 Diabetes Research Center, National Center for Global Health and Medicine, Tokyo, Japan

**Keywords:** Chronic kidney disease, Diabetic kidney disease, Surrogate endpoint, eGFR slope, Albuminuria, Proteinuria

## Abstract

**Background:**

For the development of pharmaceutical products in kidney field, appropriate surrogate endpoints which can predict long-term prognosis are needed as an alternative to hard endpoints, such as end-stage kidney disease. Though international workshop has proposed estimated glomerular filtration rate (GFR) slope reduction of 0.5–1.0 mL/min/1.73 m /year and 30% decrease in albuminuria/proteinuria as surrogate endpoints in early and advanced chronic kidney disease (CKD), it was not clear whether these are applicable to Japanese patients.

**Methods:**

We analyzed J-CKD-DB and CKD-JAC, Japanese databases/cohorts of CKD patients, and J-DREAMS, a Japanese database of patients with diabetes mellitus to investigate the applicability of eGFR slope and albuminuria/proteinuria to the Japanese population. Systematic review on those endpoints was also conducted including the results of clinical trials published after the above proposal.

**Results:**

Our analysis showed an association between eGFR slope and the risk of end-stage kidney disease. A 30% decrease in albuminuria/proteinuria over 2 years corresponded to a 20% decrease in the risk of end-stage kidney disease patients with baseline UACR ≥ 30 mg/gCre or UPCR ≥ 0.15 g/gCre in the analysis of CKD-JAC, though this analysis was not performed on the other database/cohort. Those results suggested similar trends to those of the systematic review.

**Conclusion:**

The results suggested that eGFR slope and decreased albuminuria/proteinuria may be used as a surrogate endpoint in clinical trials for early CKD (including diabetic kidney disease) in Japanese population, though its validity and cutoff values must be carefully considered based on the latest evidence and other factors.

## Introduction

Chronic kidney disease (CKD) is one of the most important factors that shortens the healthy life expectancy of the nation. Although new therapeutic drugs for CKD, such as sodium-glucose cotransporter-2 (SGLT2) inhibitors, have been developed recently, the risk of the disease remains significant, and treatment satisfaction in patients with CKD has been low. Therefore, novel drugs are required to improve the prognosis and quality of life (QOL) of patients with CKD.

To promote the early development of pharmaceutical products for CKD, methods to evaluate the clinical efficacy of therapeutic drugs are needed, that is, appropriate surrogate endpoints that predict long-term prognosis. A 30–40% reduction in the estimated glomerular filtration rate (eGFR) over 2–3 years is internationally recognized as a surrogate endpoint for advanced CKD. Our research group has analyzed data from Japanese individuals to create guidelines showing that this surrogate endpoint is appropriate [1]. However, surrogate endpoints for early CKD have not been sufficiently examined since the establishment of these guidelines. Subsequent discussions and clinical trials, mainly conducted in Europe and the United States, have led to proposals for using eGFR slope and decreased albuminuria as surrogate endpoints in early CKD [2–8].

We expect that these new surrogate endpoints will promote the development of novel therapeutic drugs for kidney diseases. Furthermore, newly approved therapeutic drugs are expected to improve the prognosis and QOL of patients and enhance public welfare, thereby contributing to achieving a healthy society with a high life expectancy and the reduction in medical expenses.

Accordingly, our research group investigated the association between long-term prognosis and eGFR slope or changes in albuminuria (following surrogate endpoints for early CKD in Europe and the United States) in the Japanese population, using databases of Japanese patients with CKD and diabetic kidney disease (DKD). It should be noted that this guideline defines CKD with eGFR of ≥ 30 mL/min/1.73 m^2^ as early CKD, regardless of the primary disease, degree of albuminuria or proteinuria.

## Preparation procedure

The progress of guideline development is summarized below. Throughout the period, discussions and exchanges of opinions were frequently conducted via email.

The first working group meeting was held on June 4, 2020, to confirm the definition of early-stage CKD, analysis methods, and role assignments of the working group members. Since then, Seiji Itano and Eiichiro Kanda conducted the analysis using a database of Japanese CKD patients (J-CKD-DB-Ex). This led to the analysis conducted by Takayuki Hamano using data from the Japanese CKD patient cohort, CKD-JAC2. Yuka Sugawara and Eiichiro Kanda led the analysis with a cohort of Japanese patients with diabetes mellitus, Japan Diabetes compREhensive database project based on an Advanced electronic Medical record System (J-DREAMS), to examine the appropriateness of the following surrogate endpoints: eGFR slope and change in albuminuria (proteinuria).

Hirokazu Okada, Koji Tomori, and Yusuke Watanabe systematically reviewed the previous papers. Kojiro Ueki and Hirotaka Watada collaborated to determine whether these results could be extrapolated to diabetic kidney disease. Masaomi Nangaku and Naoki Kashihara oversaw this analytical study. A rough draft of this guideline was prepared with Wataru Asakura based on the analytical results.

An open seminar was held on October 16, 2022, to present the draft guidelines and gather extensive views. Dick de Zeeuw (the Netherlands) was invited to this seminar as an overseas expert to discuss the differences between the trends in other countries. After revising the draft guidelines based on the seminar, public comments were gathered from the Japanese Society of Nephrology between December 5 and 18, 2022. Public comments were reviewed, and the final version of the guidelines written in Japanese was prepared and published [9]. Summarizing it, an English version of the guidelines (this article) was prepared.

## Examination of the eGFR slope as a surrogate endpoint in patients with early-stage diabetic kidney disease: Analysis using J-DREAMS

### Yuka Sugawara, Eiichiro Kanda

To evaluate whether the surrogate endpoint proposed at the 2018 National Kidney Foundation (NKF)-Food and Drug Administration (FDA)- European Medicines Agency (EMA) workshop [2] can be extrapolated to the Japanese population, we evaluated the relationship between the true endpoint, end-stage kidney disease (ESKD), and the surrogate endpoint, eGFR slope, in a population of DKD, using an epidemiological database of Japanese patients with diabetes called J-DREAMS [10].

Specifically, among patients registered in J-DREAMS, we extracted the data of 51,483 patients with “early CKD” or “no CKD” who have an eGFR record of ≥ 30 mL/min/1.73 m^2^. Subsequently, we estimated the relationship between the 1-, 2-, and 3-year eGFR slopes and the risk of ESKD (initiation of kidney replacement therapy) and composite ESKD (record of eGFR < 15 mL/min/1.73 m^2^ and kidney replacement therapy) by determining the adjusted hazard ratio (aHR) using Cox proportional hazard models. eGFR slopes were calculated using linear mixed-effects (ME) models and ordinary least-squares linear regression (OLS) models. Multiple imputations were performed for patients with missing urinary albumin-creatinine ratio (UACR) data. The details of this analysis are reported in another study [11], and a key summary is presented in this article.

**Fig. 1:**
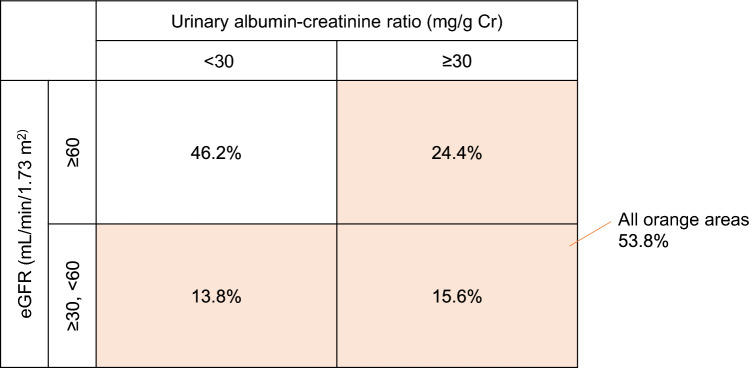
Distribution of baseline estimated glomerular filtration rate (eGFR) and urinary albumin-creatinine ratio (UACR) in the population for whom 2-year slope could be calculated in the J-DREAMS cohort. The analysis population included a significant number of patients with eGFR ≥ 60 mL/min/1.73 m^2^ or UACR < 30 mg/gCre, and only 53.8% of patients with eGFR < 60 mL/min/1.73 m^2^ or UACR ≥ 30 mg/gCre were considered to have diabetic kidney disease (orange area)

The analysis showed that, of the 12,435 patients whose 2-year slope could be calculated, 70.6% had a baseline eGFR of ≥ 60 mL/min/1.73 m^2^ and 60.0% had a normal baseline UACR (< 30 mg/g creatinine [Cr]); as a consequence, 53.8% of the total patient population was considered to have DKD, meeting the criteria of a baseline eGFR of < 60 mL/min/1.73 m^2^ and/or a baseline UACR of ≥ 30 mg/g Cr (Fig. 1). This distribution was similar for the 1- and 3-year slopes.

**Fig. 2:**
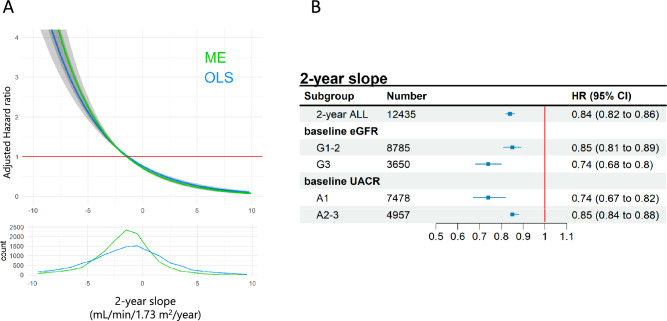
The association between the composite end-stage kidney disease (ESKD) events and 2-year estimated glomerular filtration rate (eGFR) slope. **A** Population distribution of change in eGFR slope and the association between eGFR slope and composite ESKD events. The upper panel shows the spline curve of the association between composite ESKD events and the 2-year eGFR slope. The lower panel shows the distribution of the number of cases in whom 2-year eGFR slopes were calculated. The green line corresponds to the slope calculated under the mixed-effects (ME) model, and the blue line corresponds to the slope calculated under ordinary least-squares (OLS) methods. **B** Association between composite ESKD risk and eGFR slope: subgroup analysis. Composite ESKD risk associations with eGFR slope reduction of 0.75 mL/min/1.73 m^2^/ year calculated under a ME model. eGFR slope was calculated over 2 years. HRs and 95% CIs for each subgroup divided by eGFR (G1-2: ≥ 60 mL/min/1.73 m^2^, G3: 30–60 mL/min/1.73 m^2^), or urine albumin-creatinine ratio (A1: < 30 mg/g Cr, A2-3, ≥ 30 mg/g Cr) were shown. CI confidence interval, HR hazard ratio

For any 1–3-year slopes, the aHR of composite ESKD events tended to be higher with a steeper eGFR slope in the negative direction and lower with a steeper eGFR slope in the positive direction (Fig. 2A). Similar results were obtained for both ME and OLS models; however, the correlation was slightly stronger in the ME model. Additionally, with a moderate eGFR slope at 0.75 mL/min/1.73 m^2^/year, the aHR and its 95% confidence interval (CI) of any 1–3-year slopes were below 1.

The aHRs for a reduction of 0.75 mL/min/1.73 m^2^/year in analyzing composite ESKD events were almost the same as those in analyzing ESKD events, particularly when the slope was calculated from a 1- or 2-year calculation period.

The association between the eGFR slope and risk of ESKD tended to be stronger with a longer period for the calculation of the eGFR slope. However, the sensitivity analysis, which was performed without multiple imputations for missing UACR values (listwise method), showed results different from those of the primary analysis, and we did not observe a stronger association with a longer period for the determination of the eGFR slope, thereby warranting caution.

As the baseline eGFR and UACR values were thought to affect the subsequent eGFR slope, we performed two subgroup analyses by dividing the patients by (1) baseline eGFR stage or (2) baseline UACR stage (Fig. 2B). In both subgroup analyses, the results were similar to the primary overall analysis in all groups (G1-2 group [eGFR ≥ 60 mL/min/1.73 m^2^]/G3 group [eGFR 30–60 mL/min/1.73 m^2^], and A1-2 group [UACR < 30 mg/gCr]/A3 group [UACR > 30 mg/gCr]). The relationship between composite ESKD events and eGFR slope was more evident in the G3 group than in the G1-2 group and in the A1 group than in the A2-3 group. This suggests that the potential of the eGFR slope as a surrogate endpoint for ESKD needs to be carefully considered based on the CKD stage of the population studied.

This study had a few limitations. First, this observational study used an epidemiological database; an interventional study may have yielded different results. Second, the association between eGFR slope and mortality risk could not be investigated because death in the database was optional and needed to be verified. Third, the high rate of missing UACR data may have affected the results of this study. Fourth, the mean follow-up period in this study was approximately 1 year, and patients whose ESKD events occurred after this period may not have been accurately evaluated.

In conclusion, using data from J-DREAMS, a database of Japanese patients with diabetes, we analyzed the association between the eGFR slope and the risk of ESKD. We showed an association between a reduction in eGFR slope and a reduced risk of developing ESKD observed in Japanese patients with early DKD, similar to the results presented in an overseas workshop. Our results suggest that changes in eGFR slope may be associated with the risk of developing ESKD in Japanese patients with diabetes.

## Examination of eGFR slope as a surrogate endpoint in early chronic kidney disease: Analysis using J-CKD-DB-Ex

### Seiji Itano, Eiichiro Kanda

The present study used J-CKD-DB-Ex, a database containing real-world data of patients with CKD in Japan, to evaluate whether a change in the eGFR slope, which serves as a surrogate endpoint in the early-CKD population, as proposed at the 2018 NKF-FDA-EMA workshop [2], can be extrapolated to the Japanese early-CKD population. The details of this analysis are reported in another study [12], and a key summary is presented in this article.

J-CKD-DB-Ex, the data source for this study, is a database containing chronological data on patients with CKD collected from multiple Japanese university hospitals over the years [13]. Among the registered patients, the present study examined those with two or more eGFR measurements, including baseline measurements. This study estimated the eGFR slope of patients with an eGFR of ≥ 30 mL/min/1.73 m^2^, considered patients with early CKD. The eGFR slope was calculated using OLS and ME models. The calculation periods for the eGFR slope as a surrogate endpoint were 1, 2, and 3 years. The outcome of ESKD was defined in two ways: introduction of dialysis and new-onset CKD stage G5. ESKD competes with mortality events; therefore, we performed multivariate analysis using a Fine-Gray subdistribution hazard regression model to examine the association between the eGFR slope and the sub-distribution hazard ratio (SHR) of ESKD.

The participants analyzed to calculate the eGFR slope for 1–3 years were 7,768, 6,778, and 5,219, respectively. The average observation periods of 1-, 2-, and 3-year slopes were 877 ± 491, 706 ± 346, and 495 ± 215 days. The number of deaths during the observation period was 827 (10.7%), 533 (7.9%), and 317 (6.1%), whereas the incidence of dialysis initiation was very low, with 28 (0.4%), 24 (0.4%), and 14 (0.3%) participants. The incidences of CKD stage G5 were 186 (2.4%), 129 (1.9%), and 71 (1.4%).

Regarding the distribution of the eGFR slope, the calculation of the slope from the 1-year eGFR level resulted in a larger number of patients with a significant change than the calculation from the 2- or 3-year data. This may be because 1-year data contained only a small number of eGFR measurements. Additionally, it is susceptible to short-term variability in eGFR (serum creatinine level) and short-term fluctuations due to acute renal injury and changes in muscle mass. A previous study reported that data from a follow-up period of 2–3 years were used for highly reliable calculations of the eGFR slope [2], which was also suggested by the results of the present study.

**Fig. 3:**
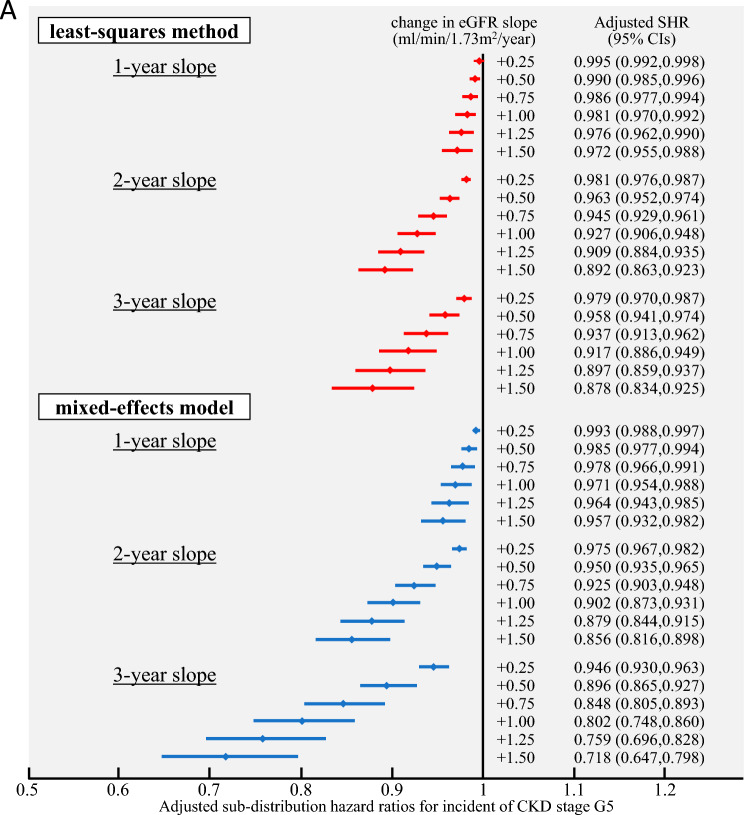

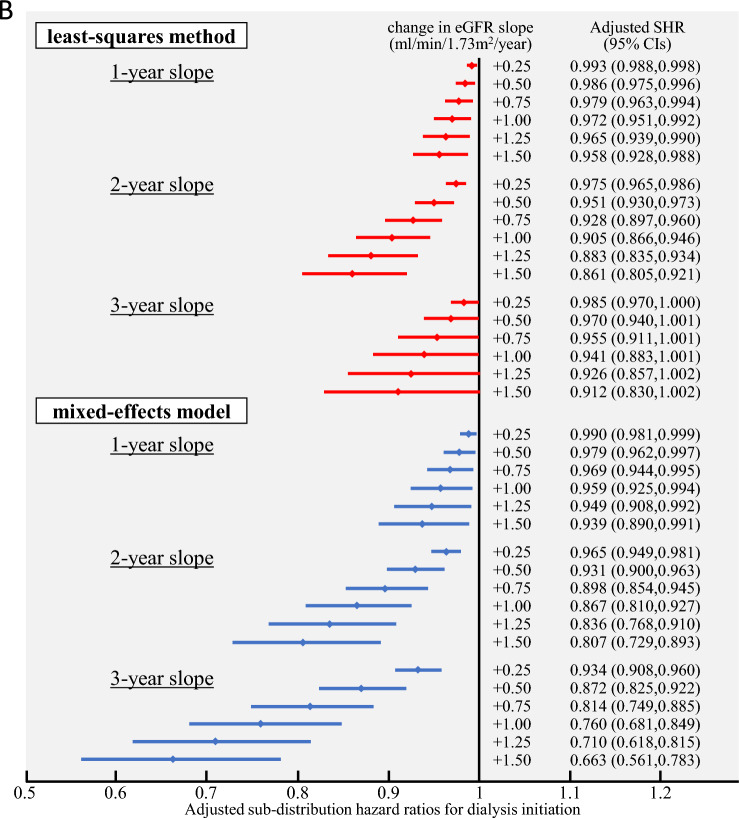
Adjusted sub-distribution hazard ratios (SHRs) for end-stage kidney disease (ESKD) occurrence by the change in estimated glomerular filtration rate (eGFR) slope. For each of the 1–3-year periods of the eGFR slope, the SHRs and 95% confidence intervals (CIs) for ESKD occurrence were shown for the range of change in eGFR slope of + 0.25 to + 1.50 ml/min/1.73 m^2^/year. **A** Adjusted SHRs for dialysis initiation and **B** adjusted SHRs for incident CKD stage G5. SHRs were estimated using a fine-gray proportional hazards regression model, with death as a competing risk. Multivariate analysis was adjusted for age, sex, eGFR, hemoglobin, serum albumin, C-reactive protein, antihypertensive medication, renin-angiotensin system inhibitor, and diabetes mellitus

Regarding the association between the 2- and 3-year eGFR slopes and ESKD development, the primary analysis, in which ESKD was defined as the introduction of dialysis, showed that the SHR (< 1) of the 3-year slope tended to decrease with an increase in the change. This was observed compared to the 2-year slope calculated using the ME model, suggesting an association between the eGFR slope and ESKD development. This tendency observed using the ME model was not observed in the eGFR slope calculated using the OLS model. Secondary analysis, in which ESKD was defined as the transition to CKD stage G5, showed that the SHR of the 3-year slope tended to exhibit a greater decrease than that of the 2-year slope in both the ME and OLS models, suggesting an association between the GFR slope and ESKD development (Fig. 3).

The limitations of this study include, first, that it was an observational study using a database based on electronic medical record information and that unmeasured confounding factors could not be considered. Second, only data from facilities in the J-CKD-DB-Ex that included information on mortality and dialysis initiation were analyzed; therefore, the external validity of the results was limited. Third, the data period of the J-CKD-DB-Ex was a maximum of 5 years, and few participants started dialysis during the observation period, except for the eGFR slope calculation period. In particular, the number of outcomes was rare when limited to participants with a baseline eGFR of > 45 mL/min/1.73 m2 or > 60 mL/min/1.73 m^2^. Further investigation is needed to determine whether similar results can be obtained when limited to an earlier CKD population. Fourth, the J-CKD-DB-Ex targets university hospitals in Japan, which are considered to have a higher severity of CKD than the general CKD population. Therefore, the renal prognosis of the analyzed population was expected to be worse than that of the general Japanese population with CKD, which may have caused a selection bias.

In conclusion, using the J-CKD-DB-Ex, a real-world database of Japanese patients with CKD, we investigated whether the relationship between a slower eGFR slope and decreased risk of ESKD, as presented in the NKF-FDA-EMA workshop also applies to patients with early CKD in Japan. Although the generalizability of this study has several limitations, it suggests that a slower eGFR slope calculated from eGFR values over 2 or 3 years is associated with a decreased risk of ESKD.

## Examination of eGFR slope and change in albuminuria/proteinuria as surrogate endpoints in chronic kidney disease—analysis of the CKD-JAC study

### Takayuki Hamano

The relevance of the eGFR slope varied depending on the baseline eGFR. The present study used data from the CKD-JAC study to analyze whether the eGFR slope and 2-year change in albuminuria or proteinuria could be used as surrogate endpoints for ESKD (dialysis introduction or transplantation) by stage.

Observational studies in Europe and the United States have suggested a slower eGFR slope by 0.5–1.0 mL/min/1.73 m^2^/year corresponds to a hazard ratio (HR) of approximately 0.7 for kidney failure with replacement therapy (KFRT). The results of the present study suggest that the longer the observation period, the stronger the association between eGFR slope and KFRT. In this study, we investigated an eGFR slope difference corresponding to a KFRT HR of 0.7–0.8; regarding the 2-year eGFR slope calculated by the ME model, a moderate difference in eGFR slope　of 0.5–0.85 mL/min/1.73 m^2^/year corresponded to a KFRT HR of 0.7–0.8 in the overall CKD population. An eGFR slope difference of approximately 0.6–0.85 mL/min/1.73 m^2^ and 0.5–0.75 mL/min/1.73 m^2^/year corresponded to this HR in CKD stage G3 and G4 patients, respectively. Therefore, the results obtained in the Japanese CKD population were broadly similar to the proposals from studies in Europe and the United States [2], these results suggest that these levels of eGFR slope difference may be used as a surrogate endpoint. However, European and United States observational studies have not been stratified according to the CKD stage. The frequency of eGFR measurements in these studies was low; several used semiannual measurements of eGFR levels. Thus, the frequency of their eGFR measurements was different from that used in the present study, which aligns with actual clinical practice in Japan. In general, the accuracy of slope evaluation increases as the number of measurements increases. Therefore, more measurements increase the possibility of using the 1-year eGFR slope as a surrogate endpoint. However, the 1-year eGFR slope difference corresponding to a certain KFRT HR would be larger than the 2-year eGFR slope difference. In addition, the association between the eGFR slope difference and KFRT is weak with an evaluation period of ≤ 1 year; thus, it may be difficult to use the eGFR slope as a surrogate endpoint with such a short evaluation period. In addition, as approximately 40% of patients with CKD stage G5 developed KFRT in 2 years in our study, it would be appropriate to use KFRT or the composite outcome of a 30% reduction in the eGFR and KFRT as an endpoint. Some randomized controlled trials (RCTs), such as those of thiazide [14] and ferric citrate [15], have been conducted in CKD stage G5; therefore, the clinical implication of the eGFR slope difference with an evaluation period of 1 year is important. This study provides recommendations for such cases. Specifically, an eGFR slope difference of approximately 1.0–1.7 mL/min/1.73 m^2^/year corresponds to a KFRT HR of 0.7–0.8 in patients with CKD stage G5 with an observation period of 1 year.

The eGFR slope calculated using the ME model showed a slightly stronger association with KFRT than that calculated using the OLS model. This is attributed to the fact that the ME model is less susceptible to outliers, is resistant to missing values, and the calculation of the eGFR slope of each individual can be partially extrapolated from the data of other patients with a similar baseline background. This was reflected in the low variability of the estimated eGFR slope in this model during the half-year period with a small number of eGFR measurements.

Observational studies in Europe and the United States have reported an association between a 30% decrease in UACR in 2 years and KFRT [2]. Additionally, an analysis using the CKD-JAC data showed that a 30% UACR decrease corresponded to a KFRT HR of 0.81. A meta-analysis by the CKD Prognosis Consortium (CKD-PC) (N = 693, 816) found that a 30% UACR decrease corresponded to an HR of 0.83 (95% CI; 0.74–0.94) [3], showing highly similar results. This meta-analysis reported that, in patients with a baseline UACR < 30 mg/g Cr, a change in UACR cannot be used as a surrogate endpoint [3]. Patients with a UACR < 30 mg/g Cr rarely develop KFRT. Most of the patients enrolled in the present study had a baseline UACR of ≥ 30 mg/g Cr. Therefore, no significant association was observed in the study population. A high amount of albuminuria induces renal tubular damage and leads to poor renal prognosis. If a decrease in UACR is the main mechanism of the benefit of an intervention on renal prognosis, a change in the UACR may be used as a surrogate endpoint in Japanese patients with a UACR of ≥ 30 mg/g Cr. Given that UACR can be measured only in patients with diabetes under medical insurance in Japan and that the urine protein-creatinine ratio (UPCR) can be covered by insurance reimbursement for all patients with CKD, it is important to determine whether UPCR change could be used as a surrogate endpoint. Overall, the strength of the correlation between UPCR change and KFRT was comparable to that of the correlation between UACR change and KFRT. However, in patients with UACR < 300 mg/g Cr, the correlation between the UPCR change and KFRT was weaker than that between the UACR change and KFRT. At low proteinuria levels, the UACR can be measured even in the range of microalbuminuria. However, measurement values of UPCR could not be obtained, especially with diluted urine, due to the detection limit of urinary protein concentration, which explains the weak correlation between UPCR and KFRT at low protein levels in our study. Although the association between the 1-year UPCR change and KFRT was slightly weaker than that between the 2-year UPCR change and KFRT, they were comparable; thus, a 1-year UPCR change could be used as a surrogate endpoint.

According to the NKF-FDA-EMA workshop, simulation studies have revealed that a combination of eGFR slope and change in UACR improves prognosis prediction. This is evident with a short observation period, such as 1 year. However, it was discussed at the workshop that with a long observation period, the eGFR slope alone can provide a certain degree of prediction, and the positive predictive value hardly improves, even when a combination of the eGFR slope and UACR change is used. This study also demonstrated that the risk of KFRT decreased with the combined use of a shallower eGFR slope and lower albuminuria. Because the association between the eGFR slope and KFRT was weak with an observation period of 1 year, the extent to which the prognostic prediction of KFRT can be improved by combining the eGFR slope with a 1-year change in UPCR remains to be investigated.

In conclusion, this study analyzed the total CKD population and targeted CKD stage G3 yielded similar results. The eGFR slope and UACR and UPCR changes may be surrogate endpoints in Japanese patients with early CKD. In the total population of this study, a moderate 2-year eGFR slope difference of approximately 0.5–0.85 mL/min/1.73 m^2^ /year corresponded to a 20–30% decrease in HR of KFRT. However, the reduction in HR varied depending on the baseline CKD stage (eGFR). In addition, a 30% decrease in the UACR over 2 years corresponded to a 20% decrease in the KFRT HR. A 30% decrease in the UPCR may also be used in the same manner as a 30% decrease in UACR. However, at a UACR of < 30 mg/g Cr and UPCR of < 0.15 g/g Cr, the association between these and KFRT was not significant, and their uses are limited.

## Review of existing evidence on surrogate endpoints in early chronic kidney disease

### Hirokazu Okada, Koji Tomori, Yusuke Watanabe

#### CQ1: Could a reduction in the eGFR slope be a surrogate endpoint for suppressing progression to end-stage kidney disease in patients with early CKD?

##### 1-1. Background

Setting appropriate surrogate endpoints in clinical trials is necessary to develop novel therapeutic drugs for CKD. As a result of the 2016 Controversies Conference on clinical trials of therapeutic drugs for renal disease, the Kidney Disease Improving Global Outcomes (KDIGO) proposed eGFR slope (incline/tilt) as one of the surrogate endpoints for early CKD (G1-G3a) with an eGFR of ≥ 45 mL/min/1.73 m^2^ and slow-progressing renal disease. In 2018, the NKF, the FDA and the EMA conducted a workshop to examine the usefulness of albuminuria and the eGFR slope as surrogate endpoints for clinical trials in the early stages of CKD. They conclude that when a reduction in the eGFR slope due to a therapeutic drug is greater than 0.5–1.0 mL/min/1.73 m^2^/year, it could be a valid surrogate endpoint to predict the suppression of progression to clinical endpoints, such as ESKD. In this context, the present review conducted the literature search for the CQ “Could a reduction in the eGFR slope be a surrogate endpoint for the suppression of progression to ESKD in patients with early CKD?”.

##### 1-2. Article selection

A literature search was performed on PubMed, targeting articles published between January 1975 and December 2021.

The search terms used were (slope)(decline)(trajectory)(glomerular filtration rate)(and end-stage renal disease)(end-stage kidney disease).

We conducted a primary review of the titles and abstracts and subsequently selected potentially relevant articles. A secondary review (full-text evaluation) was conducted to select articles relevant to CQ.

The literature search formula used was as follows:

("glomerular filtration rate"[MeSH Terms] OR ("glomerular"[All Fields] AND "filtration"[All Fields] AND "rate"[All Fields]) OR "glomerular filtration rate"[All Fields]) AND ("kidney failure, chronic"[MeSH Terms] OR ("kidney"[All Fields] AND "failure"[All Fields] AND "chronic"[All Fields]) OR "chronic kidney failure"[All Fields] OR ("end"[All Fields] AND "stage"[All Fields] AND "renal"[All Fields] AND "disease"[All Fields]) OR "end stage renal disease"[All Fields] OR ("kidney failure, chronic"[MeSH Terms] OR ("kidney"[All Fields] AND "failure"[All Fields] AND "chronic"[All Fields]) OR "chronic kidney failure"[All Fields] OR ("end"[All Fields] AND "stage"[All Fields] AND "kidney"[All Fields] AND "disease"[All Fields]) OR "end stage kidney disease"[All Fields])) AND ("slope"[All Fields] OR "sloped"[All Fields] OR "slopes"[All Fields] OR "sloping"[All Fields] OR ("decline"[All Fields] OR "declined"[All Fields] OR "decliner"[All Fields] OR "decliners"[All Fields] OR "declines"[All Fields] OR "declining"[All Fields]) OR ("trajectories"[All Fields] OR "trajectory"[All Fields] OR "trajectory s"[All Fields])).

Through this literature search, we selected articles examining whether a reduction in the eGFR slope due to treatment intervention for early CKD could serve as a surrogate endpoint that predicts the suppression of clinical endpoints, such as progression to ESKD. As a result, we selected the following articles published based on the workshop conducted by NKF, FDA, and EMA in 2018 to examine the usefulness of the eGFR slope as a surrogate endpoint for clinical trials in the early stages of CKD: (1) a meta-analysis of RCTs of treatment intervention for CKD, (2) a statistical simulation study, and (3) three articles on a meta-analysis of observational studies of CKD, as well as (4) an article examining a reduction in eGFR slope due to treatment intervention and progression to ESKD, which was published after articles (1)-(3) before December 2021.

##### 1-3. Commentary

(1) A meta-analysis of RCTs conducting treatment intervention for CKD

Inker et al. conducted a meta-analysis of 47 reported randomized controlled trials (RCTs) on treatment interventions for CKD (a total of 60,620 patients, 12 types of treatment interventions) to evaluate the eGFR slope as a surrogate endpoint for CKD progression [7]. The main treatment interventions included administering renin-angiotensin (RA) system inhibitors, strict antihypertensive therapy, protein restriction, immunosuppressive therapy, and lipid-lowering therapy. The analysis included only RCTs involving patients with a baseline eGFR of > 15 mL/min/1.73 m^2^. The mean baseline eGFR of the analysis subjects was 61.7 (standard deviation (SD) 26.4) mL/min/1.73 m^2^, and several patients with early-CKD and an eGFR of ≥ 45 mL/min/1.73 m^2^ were included. Although the analysis included a report by Katafuchi et al. targeting Japanese patients with immunoglobulin (Ig) A nephropathy and the Olmesartan-Reducing Incidence of End-stage Renal Disease in the Diabetic Nephropathy Trial (ORIENT) study, only a small number of RCTs included Japanese subjects. The mean difference in the eGFR slope (difference in the eGFR slope between the treatment intervention and control groups) and the effect of each treatment intervention on clinical endpoints (doubling of serum creatinine level, reduction in eGFR to < 15 mL/min/1.73 m^2^, and ESKD: dialysis initiation/kidney transplantation) were estimated for each RCT. For the eGFR slope, the authors calculated the total slope starting at randomization and the chronic slope starting 3 months after randomization to exclude the acute effect of treatment. They examined the association between the treatment effect on the eGFR slope and clinical endpoints using Bayesian mixed-effects analysis and investigated the degree to which the eGFR slope could predict the treatment effect on clinical endpoints.

The results showed that the 3-year eGFR total slope was – 2.94 mL/min/1.73 m^2^/year (95% CI – 3.45, – 2.43) in the treatment intervention group and – 3.49 mL/min/1.73 m^2^/year (95% CI – 4.04, – 2.93) in the control group. The chronic slope was – 3.03 mL/min/1.73 m^2^/year (95% CI – 3.49, – 2.57) in the treatment intervention group and – 3.55 mL/min/1.73 m^2^/year (95% CI – 4.07, – 3.02) in the control group. The effect of treatment intervention (difference in eGFR slope) was 0.45 mL/min/1.73 m^2^/year (95% CI 0.19, 0.72) for the 3-year total slope and 0.53 mL/min/1.73 m^2^/year (95% CI 0.32, 0.74) for the chronic slope.

The effect of reduction of treatment intervention on the 3-year eGFR total slope and chronic slope showed a strong correlation with the treatment intervention effect on the clinical endpoints ((determination coefficient R^2^ = 0.97; 95% Bayesian confidence interval (BCI) 0.78, 1.00) and (R^2^ = 0.96; 95% BCI, 0.63, 1.00)). The total slope for 1 or 2 years had a lower R^2^ value than that for ≥ 3 years. Comparing a baseline eGFR of ≥ 60 mL/min/1.73 m^2^ and eGFR of < 60 mL/min/1.73 m^2^, an eGFR of ≥ 60 mL/min/1.73 m^2^ had a higher R^2^ value than a eGFR of < 60 mlL/min/1.73 m^2^ for both the 3-year total slope and chronic slope (R^2^ = 1.00 (95% BCI, 0.87, 1.0) vs. R^2^ = 0.86 (95% BCI, 0.18, 0.99), R^2^ = 0.99 (95% BCI, 0.70, 1.00) vs. R^2^ = 0.89 (95% BCI, 0.13, 0.99)). The treatment intervention effect, with a reduction in the 3-year eGFR total slope of ≥ 0.75 mL/min/1.73 m^2^/year, reduced the hazard risk of clinical endpoints by an average of 27% (95% BCI, 20, 34). With a sufficient sample size, a treatment intervention effect with an eGFR of ≥ 0.75 mL/min/1.73 m^2^/year in the total slope for ≥ 3 years or chronic slope was thought to predict a treatment intervention effect on the progression to the clinical endpoints at a probability of at least 96%. The treatment intervention effect required to achieve a positive-reaction predictive value of 97.5% as a clinical endpoint prediction for surrogate endpoints in new future clinical trials (difference in the eGFR slope between the treatment intervention group and the control group) for a large (approximately n = 1900) and moderate sample size (approximately n = 720) was estimated to be 0.48 and 0.74 mL/min/1.73 m^2^/year for the eGFR total slope for ≥ 3 years, and 0.62 and 0.85 mL/min/1.73 m^2^/year for the chronic slope, respectively.

The authors concluded that with a sufficiently large sample size, a reduction in the eGFR slope could be a surrogate endpoint for suppressing progression to clinical endpoints in RCTs that conduct treatment interventions for CKD. They stated that a reduction in the eGFR total slope for ≥ 3 years or the chronic slope could be a surrogate endpoint for suppressing progression to ESKD, especially in early CKD with an eGFR of ≥ 60 mL/min/1.73 m^2^.

(2) Simulation study

Through simulation, Greene et al. examined the clinical trial settings in which the eGFR slope had higher statistical power compared to clinical endpoints, such as a doubling of the serum creatinine level and ESKD (reduction in eGFR to < 15 mL/min/1.73 m^2^) [5]. Time-course changes in eGFR were simulated based on data from 47 reported RCTs that conducted treatment interventions for CKD. The baseline eGFR was set by dividing the population into three groups: 27.5, 42.5, and 67.5 mL/min/1.73 m^2^. The authors examined the sample size required for appropriate statistical power based on the eGFR slopes calculated from the time of and 3 months after randomization (total slope and chronic slope, respectively). In several cases where the treatment intervention did not produce an acute effect, analysis of the eGFR slope yielded comparable or better statistical power than the clinical endpoints. In several cases, the observation period could be shortened by at least half, and the sample size could be reduced. For example, with a baseline eGFR of 42.5 mL/min/1.73 m^2^, an eGFR slope of – 3.25 mL/min/1.73 m^2^/year, and no acute effect, the use of the total slope shortened the observation period from 4–6 years to 2 years and improved the efficiency of clinical trials by 17–63% (equivalent to a reduction in sample size by 14–39%) compared to using clinical endpoints. In addition, with a baseline eGFR of 67.5 mL/min/1.73 m^2^, the statistical power of the eGFR slope increased. However, an acute effect within months of randomization may increase the risk of erroneous conclusions regarding the treatment. Furthermore, when using the total slope with an observation period of < 2 years, the presence of an acute effect reduces the efficiency of the clinical trials and increases the potential for bias. The design of clinical trials and their analyses require caution to avoid erroneous conclusions.

The authors concluded that the use of the **e**GFR slope could greatly improve the statistical power compared to the clinical endpoints, especially with a high baseline eGFR at 67.5 mL/min/1.73 m^2^ without acute effect; however, optimal endpoint setting using eGFR depends on multiple factors, such as the rate of eGFR slope, type of treatment intervention effect, and study design.

(3) Meta-analysis of observational studies of CKD

To evaluate the association between 1-, 2-, and 3-year eGFR slopes and long-term renal prognosis, Grams et al. used data from 14 observational studies on CKD-PC to perform a meta-analysis using a random-effects model [4]. The analysis included 3,758,551 subjects with a baseline eGFR of ≥ 60 mL/min/1.73 m^2^ and 122,664 subjects with an eGFR of < 60 mL/min/1.73 m^2^, with a mean observation period of 4.2 years. The results showed that for both the baseline eGFR of ≥ 60 mL/min/1.73 m^2^ and eGFR of < 60 mL/min/1.73 m^2^, a reduction in the eGFR slope of ≥ 0.75 mL/min/1.73 m^2^/year in 2 years was associated with a low risk of progression to ESKD (dialysis initiation/kidney transplantation) ([aHR 0.70; 95% CI 0.68, 0.72], [aHR 0.71; 95% CI 0.68, 0.74)). This association was weak for a 1-year eGFR slope and strong for a 3-year eGFR slope. In the patient group with a mean eGFR of 75 mL/min/1.73 m^2^ and an eGFR slope of – 5 mL/min/1.73 m^2^/year, the risk of progression to ESKD in 5 years was predicted to be 8.3%. In such a patient group with rapid progression, a treatment intervention reducing the eGFR slope to 0.75 mL/min/1.73 m^2^/year reduced the risk of progression to ESKD by 1.6% (reduced from 8.3% to 6.7%). Alternatively, in the patient group with an eGFR slope of – 1 mL/min/1.73 m^2^/year, the same treatment intervention reduced the risk of progression to ESKD by only 0.13% (reduced from 0.58% to 0.45%). The authors concluded that reducing the eGFR slope may be a superior surrogate endpoint for progression to ESKD in clinical trials, including a patient group with rapid progression.

(4) Reports examining the association between the eGFR-slope reduction due to treatment intervention for CKD and the progression to ESKD.

In the Canagliflozin and Renal Events in Diabetes with Established Nephropathy Clinical Evaluation (CREDENCE) trial, which examined the suppression effect of the SGLT2 inhibitor canagliflozin on the progression of DKD [16], the canagliflozin-administration group showed decreased progression to ESKD (reduction in eGFR to < 15 mL/min/1.73 m^2^ or dialysis initiation/kidney transplantation) by 32% [HR: 0.68; 95% CI: 0.54, 0.86; *P* = 0.002] compared to the placebo-administration group, in patients with DKD with an eGFR of 30–90 mL/min/1.73 m^2^ (mean [SD] 56.2 [18.2]) and UACR of 300–5000 mg/g Cr (median [interquartile range (IQR) 927 (463,1833)]) who had been administered with RA system inhibitors. In the CREDENCE trial, the mean eGFR slope from baseline to a median observation period of 2.62 years was – 3.19 mL/min/1.73 m^2^/year (standard error 0.15) in the canagliflozin-administration group and – 4.71 mL/min/1.73 m^2^/year (standard error 0.15) in the placebo-administration group. The difference in the eGFR slope between the two groups was 1.52 mL/min/1.73 m^2^/year (95% CI 1.11, 1.93). In addition, when the HRs of the renal composite endpoints (ESKD, doubling of serum creatinine level, and death from renal disease) were calculated using the baseline renal function (eGFR 30- < 45, 45- < 60, 60- < 90 mL/min/1.73 m^2^), the group with an eGFR of 45–60 mL/min/1.73 m^2^ showed the lowest HR, thereby indicating a renoprotective effect (HR (95% CI); eGFR 30- < 45, 0.71 (0.53–0.94); 45- < 60. 0.47 (0.31–0.72); 60- < 90, 0.81 (0.52–1.26)).

An additional analysis of the Canagliflozin Cardiovascular Assessment Study (CANVAS) Program, which investigated the suppressive effect of the SGLT2 inhibitor canagliflozin on the onset of cardiovascular events in patients with type 2 diabetes, examined its suppressive effect on renal events [17]. The canagliflozin-administration group showed significantly suppressed occurrence of renal composite endpoints [doubling of serum creatinine level, ESKD (reduction in eGFR to < 15 mL/min/1.73 m^2^ or dialysis initiation/kidney transplantation), and death from renal disease] compared to the placebo-administration group (HR 0.53; 95% CI 0.33, 0.84). In contrast, approximately 80% of the patients in this trial had a baseline eGFR of ≥ 60 mL/min/1.73 m^2^, and the number of ESKD events that occurred during the observation period was small, resulting in no significant differences in the occurrence of ESKD between the two groups (HR, 0.77; 95% CI 0.3, 1.97). The difference in the eGFR slope between the two groups was 1.2 mL/min/1.73 m^2^/year (95% CI 1.0, 1.4).

The Dapagliflozin and Prevention of Adverse Outcomes in Chronic Kidney Disease (DAPA-CKD) trial, which examined the suppression effect of the SGLT2 inhibitor dapagliflozin on CKD progression [18], included patients with CKD receiving angiotensin-converting enzyme (ACE) inhibitors or angiotensin receptor blockers (ARBs) with a baseline eGFR of 25–75 mL/min/1.73 m^2^ (mean [SD] 43.1 [12.4]) and a baseline UACR of 200–5000 mg/gCr (median 949.3). The dapagliflozin-administration group showed a decreased progression to renal composite endpoints [50% reduction in eGFR, ESKD (reduction in eGFR to < 15 mL/min/1.73 m^2^ or dialysis initiation/kidney transplantation), and death from renal disease] by 44% (HR 0.56; 95%CI, 0.45, 0.68; *P* < 0.001) compared to that in the placebo-administration group. In the DAPA-CKD trial, the eGFR slope from baseline to 2.4 years was – 2.86 mL/min/1.73 m^2^/year (SD 0.11) in the dapagliflozin-administration group and – 3.79 mL/min/1.73 m^2^/year (SD 0.11) in the placebo-administration group, and the difference in the eGFR slope between the two groups was 0.93 mL/min/1.73 m^2^/year (95% CI 0.61, 1.25). In both groups, > 40% of patients had an eGFR of ≥ 45 mL/min/1.73 m^2^, and the occurrence of primary composite endpoints (50% reduction in eGFR, ESKD, and death from renal disease or cardiovascular disease) was significantly suppressed regardless of the presence or absence of complication with type 2 diabetes.

The EMPA-REG OUTCOME trial, which examined the renoprotective effect of the SGLT2 inhibitor empagliflozin in patients with type 2 diabetes [19], compared the empagliflozin-administration (mean eGFR 74.2 mL/min/1.73 m^2^ (SD 21.6)) and placebo-administration groups (mean eGFR 73.8 mL/min/1.73 m^2^ (SD 21.1)) in patients with an eGFR of ≥ 30 mL/min/1.73 m^2^. The empagliflozin-administration group showed a decrease in renal composite endpoints (doubling of serum creatinine level, dialysis initiation/kidney transplantation, and death from renal disease) by 46% (HR 0.54; 95% CI 0.40, 0.75; *P* < 0.001). The difference in the eGFR slope between the two groups was 4.7 mL/min/1.73 m^2^ (95% CI 4.0, 5.5) at approximately 3.1 years from baseline (1.52 mL/min/1.73 m^2^/year).

The EMPA-KIDNEY trial, which examined the suppression effect of the SGLT2 inhibitor empagliflozin on CKD progression [20] (Note: Reference 19 was published outside the period covered by this literature search; however, it was included in the study as it contained an important analysis of the eGFR slope), compared the empagliflozin-administration group (mean eGFR 37.4 mL/min/1.73 m^2^ [SD 14.5]) and the placebo-administration group (mean eGFR 37.3 mL/min/1.73 m^2^ [SD 14.4]) in patients with CKD receiving RA system inhibitors with a baseline eGFR of ≥ 20 and < 45 mL/min/1.73 m^2^ or an eGFR of ≥ 45 and < 90 mL/min/1.73 m^2^ and a baseline UACR of ≥ 200 mg/g Cr. The empagliflozin-administration group showed a decrease in the renal composite endpoints (ESKD: introduction of renal replacement therapy, persistent reduction to eGFR < 10 mL/min/1.73 m^2^, persistent reduction in eGFR from baseline of ≥ 40%, and death from renal disease) by 28% (HR 0.72; 95% CI 0.64, 0.82; *P* < 0.001). The difference in the eGFR slope between the two groups was 0.75 mL/min/1.73 m^2^/year (95% CI 0.54, 0.96) for the total slope from baseline to the median observation period of 2 years and 1.37 mL/min/1.73 m^2^/year (95% CI 1.16, 1.59) for the chronic (described as “Long-Term” in the article) slope starting 2 months after the initiation of oral administration. The occurrence of renal composite endpoints was significantly suppressed, regardless of the presence or absence of diabetic complications.

Heerspink et al. reported Comparative Effectiveness of Cardiovascular Outcomes in New Users of SGLT-2 Inhibitors (CVD-REAL) 3, an international collaborative observational study, including Japan, that evaluated the renoprotective effect of SGLT2 inhibitors using a reduction in the eGFR slope, progression to ESKD (decline in eGFR to < 15 mL/min/1.73 m^2^ or initiation of dialysis/kidney transplantation), and 57%, 50%, and 40% reduction in the eGFR [21].

The study consisted of two groups of patients who were started on SGLT2 inhibitors or other antidiabetic drugs, and each patient was matched by the propensity score, such as the baseline eGFR and eGFR slope, before initiating drug administration. The administration of 71,122 new prescriptions, with 35,561 in each group, was started, and the SGLT2 inhibitors administered were dapagliflozin (57.9%), empagliflozin (34.1%), canagliflozin (5.7%), ipragliflozin (1.4%), tofogliflozin (0.5%), and luseogliflozin (0.4%). The mean baseline eGFR was 90 mL/min/1.73 m^2^, and the mean eGFR slope before initiating drug administration was – 0.73 mL/min/1.73 m^2^/year in the group receiving SGLT2 inhibitors and – 0.75 mL/min/1.73 m^2^/year in the group receiving other antidiabetic drugs. The two groups showed a difference of 1.53 mL/min/1.73 m^2^ /year (95% CI 1.34, 1.72; *P* < 0.0001) (SGLT2 inhibitors: 0.46 (0.34, 0.58) vs. other antidiabetic drugs-1.21 (– 1.35, – 1.06)) in the GFR slope during the mean observation period of 14.9 months from baseline (at start of drug administration), and a reduction in the eGFR slope due to the initiation of SGLT2 inhibitors was observed. During the mean observation period of 14.9 months, 351 cases with composite endpoints (ESKD and 50% reduction in eGFR) were noted. Overall, 114 cases (3.0/10,000 person-years) in the group received SGLT2 inhibitors and 237 cases (6.3/10,000 person-years) in the group received other antidiabetic drugs; the group receiving SGLT2 inhibitors had a significantly smaller number of composite endpoint occurrences and ESKD (HR 0.49; 95% CI 0.35, 0.67; *P* < 0.0001) (HR 0.33; 95% CI 0.16, 0.68; *P* = 0.0024).

Nagasu et al. analyzed real-world data on the renoprotective effects of SGLT2 inhibitors using a comprehensive longitudinal database of patients with CKD (J-CKD-DB-Ex) [22]. Using propensity score matching, the renoprotective effect was compared between the group that received SGLT2 inhibitors (n = 1,33) and the group that received other hypoglycemic agents (n = 1,033). The mean eGFR at the initiation of drug administration was 68.2 mL/min/1.73 m^2^ (SD 17.2) in the group receiving SGLT2 inhibitors and 68.0 mL/min/1.73 m^2^ (SD 19.1) in the group receiving other hypoglycemic agents. Comparing the total slope in the mean observation period of 21.0 months (SD 9.8) in the group receiving SGLT2 inhibitors and that in the mean observation period of 19.5 months (SD 10.4) in the group receiving other hypoglycemic agents, the eGFR slope was – 0.47 mL/min/1.73 m^2^/year (95% CI – 0.63, – 0.31) in the group receiving SGLT2 inhibitors and – 1.22 mL/min/1.73 m^2^/year (95% CI – 1.41, – 1.03) in the group receiving other hypoglycemic agents. The difference in the eGFR slope between the two groups was 0.75 mL/min/1.73 m^2^/year (95% CI 0.51, 1.00), and the group receiving SGLT2 inhibitors had a significantly reduced eGFR slope (*P* < 0.001). In addition, the number of cases progressing to composite endpoints (ESKD; reduction in eGFR to < 15 mL/min/1.73 m^2^ or 50% reduction in eGFR) was significantly smaller in the group receiving SGLT2 inhibitors than that in the group receiving other hypoglycemic agents (HR 0.4; 95% CI 0.26–0.61; *P* = 0.002).

The Finerenone in Reducing Kidney Failure and Disease Progression in Diabetic Kidney Disease (FIDELIO-DKD) trial, which examined the long-term effect of finerenone (a nonsteroidal, selective mineralocorticoid-receptor antagonist) on the renal/cardiovascular outcomes in patients with type 2 diabetes and CKD, included patients with type 2 diabetes receiving oral RA system inhibitors who corresponded to a baseline UACR of 30–300 mg/g Cr, a baseline eGFR of 25–60 mL/min/1.73 m^2^, and history of diabetic retinopathy; or a UACR of 300–5000 mg/g Cr and an eGFR of 25–75 mL/min/1.73 m^2^ (mean eGFR 44.3 mL/min/1.73 m^2^ [SD 12.6], median UACR 852 mg/g Cr [IQR: 446–1634]). During the median observation period of 2.6 years, the number of cases with the occurrence of primary composite endpoints (ESKD; reduction in eGFR to < 15 mL/min/1.73 m^2^ or dialysis initiation/kidney transplantation, persistent reduction in eGFR of ≥ 40%, and death due to renal disease) was significantly smaller in the finerenone-administration group than that in the placebo-administration group (HR 0.82; 95% CI 0.73, 0.93; *P* = 0.001) [23]. In this trial, comparing the changes in eGFR between the two groups, the finerenone-administration group demonstrated a reduction in eGFR due to acute effects after the initiation of drug administration, and the eGFR level of the finerenone-administration group crossed that of the placebo-administration group; both groups were observed to have the same eGFR level after approximately 24–28 months. The eGFR slope over 4 months after the initiation of drug administration was – 3.18 mL/min/1.73 m^2^/year (95% CI – 3.44, – 2.91) in the finerenone-administration group and – 0.73 mL/min/1.73 m^2^/year (95% CI – 1.03, – 0.44) in the placebo-administration group. The eGFR slope from 4 months after the start of drug administration to the end of the observation period was – 2.66 mL/min/1.73 m^2^/year (95% CI – 2.96, – 2.36) in the finerenone-administration group and – 3.97 mL/min/1.73 m^2^/year (95% CI – 4.27, – 3.66) in the placebo-administration group.

##### 1–4 Summary

A review of summarized articles presented at a workshop conducted by the NKF, FDA, and EMA in 2018 examined the usefulness of albuminuria and the eGFR slope as surrogate endpoints for clinical trials in early CKD. The review stated that when the reduction in the eGFR slope due to therapeutic drugs in early CKD with eGFR of ≥ 45 mL/min/1.73 m^2^ is greater than 0.5–1.0 mL/min/1.73 m^2^/year over a mean observation period of 2–3 years, it can be a valid surrogate endpoint equivalent to an eGFR reduction of > 40% in phase 3 clinical trials of therapeutic drugs for CKD [2]. In addition, multiple RCTs and observational studies examining the suppressive effect of the above SGLT2 inhibitors on renal events (Table 1) have shown that therapeutic drugs that reduce the eGFR slope by approximately 0.75–1.53 mL/min/1.73 m^2^/year in patients with early CKD reduce the risk of progression to clinical endpoints. Additionally, a reduction in the eGFR slope greater than at least 0.75 mL/min/1.73 m^2^/year may serve as a surrogate endpoint for suppressing progression to ESKD. However, some therapeutic drugs exhibit an acute effect after their administration, and the degree of eGFR reduction due to the acute effects and the recovery period from the acute effect varies depending on the drug.

**Table 1:** Abstract table

Trial	CREDENCE [16]	CANVAS Program [17]	DAPA-CKD [18]	EMPA-REG OUTCOME [19]	EMPA-KIDNEY [20]	CVD-REAL 3 [21]	Nagasu, Kashihara (J-CKD-DB-Ex) [22]	FIDELIO-DKD [23]
Subjects	DKD	Type 2 DM	CKD	Type 2 DM	CKD	Type 2 DM	CKD & Type 2 DM	CKD & Type 2 DM
Drug	Canagliflozin	Canagliflozin	Dapagliflozin	Empagliflozin	Empagliflozin	Six SGLT2I^A^	Six SGLT2I^A^	Finerenone
Baseline eGFR, ml/min/1.73 m^2^, mean ± SD	56.2 ± 18.2	76.5 ± 20.5	43.1 ± 12.4	74.0 ± 21.4	Empagliflozin 37.4 ± 14.5 Placebo 37.3 ± 14.4	SGLT2I 90.6 ± 21.5 Other HGA 90.9 ± 23.1	SGLT2I 68.2 ± 17.2 Other HGA 68.0 ± 19.1	44.3 ± 12.6
Baseline UACR, mg/gCr, median (IQR)	927 (463–1833)	12.3 (6.65–42.1)	Dapagliflozin 965 (472–1903) Placebo 934 (482–1868)	Placebo < 30: 59.2%, 30–300: 28.9%, > 300: 11.1% Empagliflozin < 30: 59.5%, 30–300: 28.5%, > 300: 10.9%	Empagliflozin 331 (46–1061) Placebo 327 (54–1074)	No data	Presence of proteinuria SGLT2I 28.5% Other HGA 27.5%	852 (446–1634)
Observation period, years	2.62 (median)	2.63 (median)	2.4 (median)	3.1 (median)	2.0 (median)	1.24 (mean)	SGLT2I 1.75 Other HGA 1.63 (mean)	2.6 (median)
Type of eGFR slope	Total	Total	Total	Total	Total & chronic^B^	Total	Total	Chronic^C^
Difference in eGFR slope, ml/min/1.73 m^2^/year (95% CI)	1.52 (1.11, 1.93)	1.2 (1.0, 1.4)	0.93 (0.61, 1.25)	1.52 (1.29, 1.77)	Total 0.75 (0.54, 0.96) Chronic 1.37 (1.16, 1.59)	1.53 (1.34, 1.72)	0.75 (0.51, 1.0)	1.31
HR for clinical endpoint (95% CI)	0.68 (0.54, 0.86)	0.77 (0.3, 1.97)	0.56 (0.45, 0.68)	0.54 (0.40, 0.75)	0.72 (0.64, 0.82)	0.49 (0.35, 0.67)	0.4 (0.26, 0.61)	0.82 (0.73, 0.93)

CI, confidence interval; CKD, chronic kidney disease; DKD, diabetic kidney disease; DM, diabetes mellitus; HGA, hypoglycemic agents; SGLT2I, sodium glucose cotransporter-2 inhibitors

^A^Six types of SGLT2I includes dapagliflozin, empagliflozin, canagliflozin, ipragliflozin, tofogliflozin, and luseogliflozin

^B, C^Chronic slope starts (B) 2 months and (C) 4 months after the initiation of drug administration

In conclusion, we propose that “a reduction in the eGFR slope could be a surrogate endpoint for the suppression of progression to ESKD in patients with early-stage CKD.” A cutoff value of ≥ 0.5–1.0 mL/min/1.73 m^2^/year is considered a guide when a reduction in the eGFR slope is used as a surrogate endpoint in the clinical trials of novel CKD therapeutic drugs. Regarding the selection of evaluation methods for the eGFR slope (the total slope, which is evaluated from baseline, or chronic slope, which is evaluated after the occurrence of acute effects), it was stated that the total slope is the basis, owing to the fact that the presence and degree of acute effects and the recovery period from acute effects may not be predictable in advance, and the results of multiple RCTs and observational studies using SGLT2 inhibitors. For setting the evaluation period, when using the total slope with an observation period of < 2 years, an acute effect reduces the efficiency of a clinical trial and increases the potential for bias and when using the total slope, the treatment effect may be underestimated in clinical trials of therapeutic drugs with a long-lasting acute effect. Considering these, an observation period of 2–3 years or longer is considered desirable.

#### CQ2: Can a decrease in albuminuria/proteinuria serve as a surrogate endpoint for the suppression of progression to end-stage kidney disease in patients with early CKD?

##### 1-1. Background

Albuminuria and proteinuria are independent risk factors of ESKD [24–26]. Several reports have suggested its direct involvement in the progression of renal injury, and it is one of the most reproducible and clinically valuable biomarkers of CKD [27]. However, whether this change in albuminuria/proteinuria can be used as a surrogate endpoint for intervention trials in early CKD remains controversial [28, 29].

In March 2018, the NKF, FDA, and EMA jointly held a workshop to examine the validity of albuminuria/proteinuria and changes in the eGFR as surrogate endpoints of early CKD [2]. We concluded that an early change in albuminuria may be a valid surrogate endpoint for CKD progression, although its appropriateness varies depending on the primary disease and intervention. In this context, the present review searched the literature for the CQ “Can a decrease in albuminuria/proteinuria serve as a surrogate endpoint for the suppression of progression to ESKD?”.

##### 1-2. Article selection

Using the search formula below, a comprehensive literature search was conducted using PubMed, targeting articles published between January 1, 2016, and December 31, 2021. After selecting the potentially relevant articles, we conducted a secondary review (full-text evaluation) of the titles and abstracts of 974 articles. We manually searched the articles and selected those relevant to this CQ.

The literature search formula used was as follows:

(("kidney failure, chronic"[MeSH Terms] OR ("kidney"[All Fields] AND "failure"[All Fields] AND "chronic"[All Fields]) OR "chronic kidney failure"[All Fields] OR ("end"[All Fields] AND "stage"[All Fields] AND "kidney"[All Fields] AND "disease"[All Fields]) OR "end stage kidney disease"[All Fields] OR ("kidney failure, chronic"[MeSH Terms] OR ("kidney"[All Fields] AND "failure"[All Fields] AND "chronic"[All Fields]) OR "chronic kidney failure"[All Fields] OR ("end"[All Fields] AND "stage"[All Fields] AND "renal"[All Fields] AND "disease"[All Fields]) OR "end stage renal disease"[All Fields]) OR ("kidney failure, chronic"[MeSH Terms] OR ("kidney"[All Fields] AND "failure"[All Fields] AND "chronic"[All Fields]) OR "chronic kidney failure"[All Fields] OR ("chronic"[All Fields] AND "renal"[All Fields] AND "failure"[All Fields]) OR "chronic renal failure"[All Fields]) OR ("renal insufficiency, chronic"[MeSH Terms] OR ("renal"[All Fields] AND "insufficiency"[All Fields] AND "chronic"[All Fields]) OR "chronic renal insufficiency"[All Fields] OR ("chronic"[All Fields] AND "renal"[All Fields] AND "insufficiency"[All Fields])) OR ("renal insufficiency, chronic"[MeSH Terms] OR ("renal"[All Fields] AND "insufficiency"[All Fields] AND "chronic"[All Fields]) OR "chronic renal insufficiency"[All Fields] OR ("chronic"[All Fields] AND "kidney"[All Fields] AND "disease"[All Fields]) OR "chronic kidney disease"[All Fields])) AND ("albuminuria"[MeSH Terms] OR "albuminuria"[All Fields] OR ("proteinuria"[MeSH Terms] OR "proteinuria"[All Fields] OR "proteinurias"[All Fields]))) AND ((clinicalstudy[Filter] OR clinicaltrial[Filter] OR meta-analysis[Filter] OR multicenterstudy[Filter] OR observationalstudy[Filter] OR randomizedcontrolledtrial[Filter] OR systematicreview[Filter]) AND (2016/1/1:2020/12/31[pdat]))

At the NKF-FDA-EMA workshop mentioned above, the validity of surrogate endpoints in clinical trials of novel drugs for early CKD was examined, and previously published evidence was summarized and analyzed [2]. Therefore, we selected the following articles as evidence for the CQ: 1. an article on the meta-analysis of the CKD-PC cohort [3], and 2. an article on the meta-analysis of RCTs [6], which was presented at the workshop and published in Lancet Diabetes and Endocrinology in 2019, as well as four articles on the *post-hoc* studies of RCTs of SGLT-2 inhibitors and glucagon-like peptide-1 (GLP-1) analogs [30–33], an article on an RCT of a mineralocorticoid receptor (MR) antagonist [23], and an article on a meta-analysis on IgA nephropathy [34], which were published after the workshop.

##### 1-3. Commentary

1. Meta-analysis of the CKD-PC cohort study

Coresh et al. extracted the data of 693,816 patients from 28 cohorts with CKD-PC and examined the association between the change in baseline 2-year UACR or UPCR and the subsequent risk of developing ESKD [3]. The overall patient demographics were as follows: mean age, 63 years; female, 25%; diabetes, 80%; eGFR, 78 mL/min/1.73 m^2^. The baseline UACR or UPCR varied according to the baseline eGFR. The results showed that 7,461 patients developed ESKD (initiation of renal replacement therapy), and the change in UACR or UPCR was associated with the risk of ESKD, with a UACR decrease of ≥ 30% in 2 years leading to a decrease in the ESKD risk by 22% (HR 0.78; 95% CI 0.66, 0.92). This effect was comparable between patients with and without diabetes. However, the effect was greater with a higher baseline UACR, and at a baseline UACR of ≥ 300 mg/g, a 30% decrease in UACR in 2 years was estimated to reduce the absolute risk of ESKD by ≥ 1% in 10 years. Similar results were obtained with a change in the UPCR.

(2) Meta-analysis of RCTs

To clarify the association between the treatment effect on early changes in UACR and the treatment effects on clinical endpoints, Heersplink et al. conducted a meta-analysis of RCTs evaluating the effects of six treatment interventions (RA system inhibitors, intensive antihypertensive therapy, protein restriction, immunosuppressive agents, sulodexide, and SGLT2 inhibitors) [6]. They extracted the data of 29,973 patients from 41 trials and evaluated the validity of the change in UACR as a surrogate endpoint for CKD progression by modeling the association between the treatment effects on the change in UACR over 6 months and those on the clinical endpoints. The overall patient demographics were as follows: mean age, 58.2 years; female, 33.2%; diabetes, 70.7%; baseline eGFR, 58.2 mL/min/1.73 m^2^; and UACR, 272 mg/gCr. With a median follow-up period of 3.4 years, renal composite endpoints (ESKD, eGFR < 15 mL/min/1.73 m^2^, and doubling of the serum creatinine level) occurred in 3,395 patients (13.1%). The treatment effect on UACR was associated with clinical endpoints (R^2^ = 0.47; 95% BCI, 0.02, 0.96), and a 30% decrease in the UACR reduced hazard risk for renal composite endpoints by an average of 27% (95% BCI, 5, 45). In addition, the population with a high baseline UACR (UACR > 30 mg/g Cr) exhibited a stronger correlation between the decrease in UACR and treatment effect on clinical endpoints (R^2^ = 0.72; 95% BCI, 0.05, 0.99). Using a prediction model, the threshold for a UACR decrease over 6 months, with an assurance of usefulness of 97.5% for clinical endpoints in a randomized controlled trial, was estimated to be a 21% decrease in a large-scale trial (approximately n = 1,000) and a 27% decrease in a medium-scale trial (approximately n = 200).

Based on the results of the phase 3 RCTs, the NKF-FDA-EMA workshop concluded that an early change in UACR may be a reasonable surrogate endpoint for the progression of renal disease, depending on the circumstances.

(3) Studies reported after the NKF-FDA-EMA workshop

*Post-hoc* study of the CREDENCE trial

A *post-hoc* study of the CREDENCE trial, which examined the renal outcomes with canagliflozin in a placebo-controlled manner in patients with type 2 diabetes and overt albuminuria (UACR > 300 mg/gCr) [n = 3,836 (Asian 19.9%), eGFR 56.2 ml/min/1.73 m^2^ (SD 18.2). UACR 927 mg/gCr (IQR, 463–1,833)] evaluated whether the effect of canagliflozin on UACR and early changes in UACR (rate of change over 26 weeks) was associated with the main renal composite outcomes (ESKD, doubling of serum creatinine level, and death from renal disease) [30]. The results showed that UACR in the canagliflozin group decreased by 31% (95% CI 27, 36) after 26 weeks compared to that in the placebo group, and the canagliflozin group was significantly more likely to achieve a UACR decrease of ≥ 30% (odds ratio (OR) 2.69; 95% CI 2.35, 3.07). An early change in the UACR (rate of change over 26 weeks) and renal composite outcomes showed a log-linear association, and each 30% decline in UACR resulted in a 29% reduction in the hazard risk for renal composite outcomes (HR 0.71, 95% CI 0.67, 0.76). Therefore, an early change in UACR was also shown to be independently associated with long-term renal and cardiovascular outcomes, even with SGLT2 inhibitors.

*Post-hoc* study of the EMPA-REG OUTCOME trial

A *post-hoc* study of the EMPA-REG OUTCOME trial, which evaluated the cardiovascular and renal risks of empagliflozin in 7,028 patients with type 2 diabetes and cardiovascular disease (mean age [SD], 63.1 years [8.6]; female, 28.5%; eGFR 74.1 mL/min/1.73 m^2^ [SD 21.3]; UACR 17.7 mg/g Cr [IQR 6.2–71.6]), adopted the rate of change from baseline to week 12 as the initial change in UACR [31]. The results showed that empagliflozin decreased the UACR by 18% (95% CI 14, 22) at week 12 compared to placebo and significantly increased the likelihood of having a UACR decrease of ≥ 30% (OR 1.42; 95% CI 1.27, 1.58). During the 3.0 years of the follow-up period, 168 renal composite events (40% reduction in eGFR, eGFR < 15 mL/min/1.73 m^2^, introduction of renal replacement therapy, and death due to renal disease) were observed. As a continuous variable, the association between the change in UACR at week 12 and renal composite events was evaluated, and each 30% decline in UACR at week 12 resulted in an average reduction of 17% in renal composite outcomes (HR 0.83; 95% CI 0.78, 0.89; *P* < 0.001). An early change in UACR after the initiation of empagliflozin administration was concluded to be associated with long-term cardiovascular and renal risks and that early changes in UACR in patients administered empagliflozin may be a useful marker for predicting the prognosis of renal disease.

Prespecified analysis of the DAPA-CKD trial

The DAPA-CKD trial examined the effect of dapagliflozin on the renal and cardiovascular outcomes in 4,304 patients with CKD (baseline eGFR 25–75 mL/min/1.73 m^2^ and baseline UACR 200–5,000 mg/gCr) with or without type 2 diabetes (mean age, 61.8 years; female, 33.1%; type 2 diabetes, 67.5%; eGFR, 43.1 mL/min/1.73 m^2^; UACR, 949 mg/gCr). A prespecified analysis of the DAPA-CKD trial evaluated the effect of dapagliflozin on UACR and the association between an early change in UACR and long-term change in renal function [32]. The results showed that the geometric mean rate of change in UACR by dapagliflozin compared to that by placebo was – 26.5% (95% CI – 30.9, – 22.1; *P* < 0.0001) at week 2 and – 29.3% (95% CI – 33.1, – 25.2; *P* < 0.0001) over the entire follow-up period. This effect was – 14.8% (95% CI – 22.9, – 5.9; *P* = 0.0016) in nondiabetic nephropathy patients and – 35.1% (95% CI – 39.4, – 30.6; *P* < 0–0001) in diabetic nephropathy patients, showing a greater effect in patients with diabetes. In addition, an examination of the change in UACR from baseline over 2 weeks after the start of the trial and the rate of subsequent reduction in eGFR revealed an inverse correlation: the rate of reduction in eGFR over time decreased with a greater decrease in UACR at week 2 (β = –3.06, *P* = 0.0056). This association was consistent in both patients with (β = – 2.78, *P* < 0.0001) and without diabetes (β = – 3.35, *P* < 0.0001). The association between an early decrease in UACR and the long-term suppression of eGFR reduction suggests the importance of monitoring UACR as a marker to guide patient management.

*Post-hoc* study of the effect and action of liraglutide in diabetes: evaluation of cardiovascular outcome results (LEADER) trial

A *post-hoc* study of the LEADER trial, which examined the long-term effects of liraglutide on cardiovascular outcomes in patients with type 2 diabetes at high risk for cardiovascular disease, analyzed the 1-year change in UACR and the risk for subsequent renal composite outcomes (doubling of serum creatinine level, eGFR < 45 mL/min/1.73 m^2^, introduction of renal replacement therapy, and death from renal disease) in trial participants (8270 patients) [33]. Among the subgroups with microalbuminuria or overt albuminuria at baseline, the group with a UACR decrease of ≥ 30% had a significantly decreased number of renal composite events (HR 0.67; 95% CI 0.49, 0.93). It was concluded that a decrease of ≥ 30% in the rate of UACR after 1 year may be associated with improvement in renal prognosis.

An RCT examining the effect of finerenone in patients with DKD (FIDELIO-DKD trial).

Bakris et al. examined the long-term effects of finerenone, a nonsteroidal, selective mineralocorticoid-receptor antagonist, on the renal/cardiovascular outcomes in 5,674 patients with CKD complicated with type 2 diabetes [mean age, 65.6 years; male, 70.2%; mean eGFR (SD), 44.3 mL/min/1.73 m^2^ (12.6); median UACR (IQR), 852 mg/gCr (446, 1634) [23]. With a median observation period of 2.6 years, primary composite endpoints (ESKD, persistent reduction in eGFR of ≥ 40%, and death from renal disease) occurred in 504 patients (17.8%) in the finerenone group and 600 patients (21.1%) in the placebo group, showing their significantly less occurrence in the finerenone group (HR 0.82; 95% CI 0.73, 0.93; *P* = 0.001). In addition, the finerenone group showed a 31% decrease in the UACR from the baseline to month 4 compared with the placebo group.

Meta-analysis of RCTs for IgA nephropathy

Inker et al. conducted a meta-analysis of 12 RCTs with data from 1037 patients examining four types of interventions for IgAN (RA system inhibitors, fish oil, and other immunosuppressive agents) and evaluated the association between the treatment effect on the early change in UPCR and the treatment effect on the eGFR slope [34]. They extracted the data of 1,037 patients from 12 trials (mean age, 39.7 years (SD 12.5); female, 33.5%; mean eGFR, 71.9 mL/min/1.73 m^2^ (SD 29.8); UPCR 1.8 mg/g Cr) and modeled the association between the treatment effect on UPCR (measured at baseline and 6 months) and that on the eGFR slope (total slope from baseline or chronic slope from 3 months after randomization). The mean rate of change in UPCR over 6 months was – 35% (IQR, − 57, 18) in the control group and – 53% (IQR, − 68, − 9) in the treatment group, showing a treatment effect corresponding to a geometric mean ratio of 0.75 (95% CI 0.61, 0.94). The mean treatment effect on the 3-year total slope (1.39 mL/min/1.73 m^2^/year [95% CI – 0.21, 2.99]) was stronger than the mean treatment effect on the chronic slope (0.70 mL/min/1.73 m^2^/year [95% CI – 0.62, 2.02]), although a variation between studies was noted. Regarding the association between the treatment effect on UPCR and the treatment effect on the eGFR slope, all studies showed a significant association between the treatment effect on UPCR at 6 months, the treatment effect on the total slope after 3 years (median R^2^ = 0.88; 95% BCI, 0.06, 1), and treatment effect on the chronic slope (R^2^ = 0.98; 95% BCI, 0.29, 1). With a 30% decrease in UPCR after 6 months, the probability that the treatment effect on the total slope after 2 or 3 years and the chronic slope would not be zero was estimated to be approximately 90%. These results suggest that an early decrease in UPCR may be used as a surrogate endpoint in studies on IgA nephropathy progression.

##### 1-4. Summary

As with the eGFR slope, the evidence for a “decrease in albuminuria/proteinuria” published previously was summarized and analyzed at the NKF-FDA-EMA workshop [2]. It was concluded that a UACR decrease of ≥ 30% over 6 months in patients with CKD with a UACR of ≥ 30 mg/gCr could serve as a surrogate endpoint for the progression to ESKD. Since the workshop, the CREDENCE trial using SGLT2 inhibitors, a *post-hoc* study of the EMPA-REG OUTCOME trial, a prespecified analysis of the DAPA-CKD trial, and a *post-hoc* study of the LEADER trial on GLP-1 analogs have been reported. Additionally, all interventions showed that the rate of early change in UACR was associated with renal outcomes and that a 30% decrease in UACR resulted in a significant reduction in HR. The FIDELIO-DKD trial with the MR antagonist, finerenone, also showed a decrease in UACR and improvement in renal outcomes. Furthermore, a meta-analysis of RCTs for IgA nephropathy reported an association wherein the eGFR slope is reduced with a greater decrease in UACR, showing that with a 30% decrease in the UPCR over 6 months, the treatment effect on the eGFR slope was predicted with an accuracy of ≥ 90% [29]. These results suggest that a UACR decrease of ≥ 30% over 6 months may serve as a surrogate endpoint in clinical trials, especially in intervention trials of diseases with UACR and drugs, whose main pharmacological action is thought to be a decrease in UACR.

## Summary

Although drawing a definite conclusion is difficult, the results of observational studies using various databases conducted by our research group, as well as meta-analyses and observational studies reported overseas, suggest that the eGFR slope may be used as a surrogate endpoint in some clinical trials for early CKD (including DKD). However, its validity and cutoff values must be carefully considered based on the latest evidence, primary disease, target population, drug characteristics, and other factors.

The eGFR slope may be used as a surrogate endpoint if the subjects of the clinical trial comprise a relatively homogeneous population (such as a population with a relatively narrow eGFR range and known histology and progression of chronic glomerulonephritis) and if the effect of a drug is assumed to be constant over the years. The eGFR slope generally assumes the total slope from the initiation of drug administration, including the drugs that reduce eGFR during the early administration (so-called “initial drop”). The validity of setting the eGFR slope should be considered based on drug characteristics. In addition, considering the pathology of early CKD, it is desirable to evaluate the eGFR slope based on an observation period of at least 2 years. Sufficient consideration is needed for the specific setting of the cutoff value of the eGFR slope. Generally, the higher the cutoff value, the stronger the evidence obtained. The eGFR-slope cutoff value of 0.5–1.0 mL/min/1.73 m^2^/year is assumed, as proposed in discussions in Europe and the United States, can be used in some cases. However, depending on the baseline eGFR, the slope values may be within the range of intra-individual variation. Using indices according to the primary disease and characteristics of drugs in setting the verification hypothesis is necessary; therefore, consultation with the Pharmaceuticals and Medical Devices Agency (PMDA) on trial plans (including the use of the eGFR slope and its cutoff value) and interpretation of trial results are strongly recommended for clinical trials (therapeutic testing) aimed at regulatory approval.

Regarding albuminuria (UACR) and proteinuria (UPCR), observational studies conducted by our research group suggested that a decrease of ≥ 30% from the baseline over approximately 1 or 2 years is a guide in patients with a UACR of 30 mg/g Cr or UPCR of ≥ 150 mg/g Cr. Generally, the higher the cutoff value, the stronger the evidence obtained. Using indices according to the primary disease and characteristics of drugs in setting the verification hypothesis is necessary; therefore, consultation with the PMDA on trial plans (including the use of UACR and its cutoff value) and interpretation of trial results are strongly recommended for clinical trials (therapeutic testing) aimed at regulatory approval.

In addition, regarding the surrogate endpoints in clinical trials for early CKD, the content presented in this article is based on the findings obtained thus far. Further evidence and reviews are required in the future.
